# Engineering probiotic consortia to improve chemotherapy tolerance and reduce toxicity in breast cancer patients: a new frontier in supportive precision oncology

**DOI:** 10.3389/or.2026.1909404

**Published:** 2026-09-08

**Authors:** Emmanuel Ifeanyi Obeagu

**Affiliations:** 1 Department of Biomedical and Laboratory Science, Africa University, Mutare, Zimbabwe; 2 Department of Molecular Medicine and Haematology, University of the Witwatersrand, Johannesburg, South Africa; 3 Department of Haematology and Immunohaematology, Bahir Dar University, Bahir Dar, Ethiopia

**Keywords:** breast cancer, chemotherapy tolerance, gut microbiome, mucosal toxicity, probiotic consortia

## Abstract

Chemotherapy is a cornerstone of breast cancer treatment but is often limited by systemic toxicities, gastrointestinal mucositis, immunosuppression, and treatment interruptions, which compromise patient outcomes. Emerging evidence highlights the gut microbiome as a central mediator of chemotherapy tolerance and toxicity. Cytotoxic regimens induce dysbiosis, characterized by depletion of beneficial commensals, expansion of pathobionts, and impaired microbial metabolic function, exacerbating mucosal injury, inflammation, and systemic side effects. Engineered probiotic consortia—rationally designed multi-strain microbial communities—offer a novel strategy to restore microbial balance, reinforce epithelial barrier function, modulate immune responses, and enhance chemotherapy tolerance. Preclinical and emerging clinical studies demonstrate that multi-strain consortia can increase short-chain fatty acid production, reduce pro-inflammatory signaling, preserve mucosal integrity, and improve patient resilience during treatment. Advances in synthetic biology and microbiome engineering enable precise design of microbial consortia with complementary functions and ecological stability. This review provides a comprehensive overview of the mechanistic rationale, preclinical and clinical evidence, technological strategies, and translational implications of engineered probiotic consortia, highlighting their potential to transform supportive care in breast cancer therapy.

## 1 Introduction

Breast cancer remains one of the most prevalent malignancies worldwide, with chemotherapy serving as a foundational component of both adjuvant and neoadjuvant treatment regimens. While cytotoxic agents such as anthracyclines, taxanes, cyclophosphamide, and fluoropyrimidines have demonstrated robust antitumor efficacy, their clinical utility is frequently constrained by systemic and gastrointestinal toxicities (1, 2). Chemotherapy-induced mucositis, immunosuppression, fatigue, and metabolic disturbances are among the most common complications, adversely affecting patient quality of life, nutritional status, and adherence to treatment protocols. These toxicities can lead to dose reductions, treatment delays, or discontinuation, ultimately impacting therapeutic outcomes and long-term prognosis (3, 4). Recent research has increasingly highlighted the gut microbiome as a central modulator of chemotherapy efficacy and toxicity. The gut microbiota, composed of trillions of bacteria, fungi, and viruses, maintains gastrointestinal homeostasis through multiple mechanisms, including epithelial barrier support, regulation of mucosal immunity, metabolic processing of nutrients, and modulation of systemic inflammatory responses. Chemotherapy disrupts this ecosystem, inducing dysbiosis characterized by reduced microbial diversity, depletion of beneficial commensals such as *Lactobacillus*, Bifidobacterium, Faecalibacterium prausnitzii, and Akkermansia muciniphila, and expansion of opportunistic pathobionts including *Enterococcus* and Enterobacteriaceae. These microbial shifts exacerbate mucosal injury, impair epithelial repair, promote inflammatory cascades, and may influence drug metabolism and systemic toxicity (5, 6).

Traditional probiotic strategies have demonstrated some success in mitigating chemotherapy-induced gastrointestinal toxicity, particularly single-strain *Lactobacillus* or Bifidobacterium formulations. However, these approaches often fail to replicate the complexity and functional redundancy of a healthy microbiome. Single strains are limited in their ability to restore ecological balance, provide complementary metabolic functions, and counteract the multifaceted impacts of chemotherapy-induced dysbiosis. This limitation has prompted the exploration of engineered probiotic consortia, in which multiple microbial strains are rationally combined to provide synergistic and complementary benefits. Such consortia can collectively restore short-chain fatty acid (SCFA) production, reinforce mucosal barrier integrity, modulate inflammatory and immune pathways, and competitively inhibit pathogenic overgrowth (7, 8).

Advances in synthetic biology, microbial ecology, and systems biology now enable the design of consortia with defined functional capabilities, ecological stability, and targeted therapeutic potential. Rationally engineered consortia can be tailored to the specific chemotherapy regimen, baseline microbial composition, and immune status of individual patients, allowing for a precision-based approach to supportive care. Beyond gastrointestinal protection, these interventions have the potential to influence systemic immune responses, reduce oxidative stress, and enhance overall treatment tolerance, ultimately improving patient outcomes and quality of life (9, 39). This review aims to provide a comprehensive overview of the current state of knowledge regarding engineered probiotic consortia in breast cancer therapy. It will examine the pathophysiological interplay between chemotherapy and gut microbiome dysbiosis, summarize mechanistic and preclinical evidence supporting multi-strain probiotic strategies, explore technological and translational advances in microbiome engineering, and discuss clinical implications and future directions for integrating rationally designed probiotic consortia into supportive oncology care.

Accumulating evidence indicates that the gut microbiome plays a fundamental role in breast cancer development, progression, and therapeutic response, providing an important biological rationale for microbiome-targeted supportive interventions. The intestinal microbial community regulates host immunity, systemic inflammation, energy metabolism, and endocrine homeostasis, all of which influence breast tumor biology. One of the most extensively studied mechanisms involves the estrobolome, the collection of gut microbial genes capable of metabolizing estrogens through β-glucuronidase activity. By regulating the deconjugation and enterohepatic recirculation of estrogens, the estrobolome can alter circulating estrogen concentrations, potentially influencing the risk and progression of hormone receptor-positive breast cancer (39). Furthermore, microbial-derived metabolites, including short-chain fatty acids, secondary bile acids, indole derivatives, and other bioactive compounds, modulate epithelial integrity, immune cell differentiation, and inflammatory signaling pathways that contribute to tumor initiation and progression. Dysbiosis has been associated with impaired antitumor immunity, chronic activation of pro-inflammatory cytokines, reduced microbial diversity, and altered responses to chemotherapy and immunotherapy. Chemotherapeutic agents can further exacerbate microbial imbalance, creating a bidirectional interaction in which treatment-induced dysbiosis contributes to toxicity while the pre-existing microbiome influences therapeutic efficacy (9). Consequently, strategies aimed at restoring or engineering beneficial microbial communities have emerged as promising approaches to improve treatment tolerance, preserve intestinal homeostasis, and optimize clinical outcomes. These observations provide a compelling biological framework for the development of engineered probiotic consortia as an innovative component of supportive precision oncology for patients with breast cancer.

## 2 Aim

This review aims to provide a comprehensive synthesis of current knowledge on the role of engineered probiotic consortia in improving chemotherapy tolerance and reducing toxicity in breast cancer patients. It seeks to elucidate the mechanistic interplay between chemotherapy-induced dysbiosis, epithelial injury, and systemic inflammation, and to evaluate emerging strategies that leverage multi-strain microbial communities to restore microbial balance, support mucosal integrity, and modulate immune responses.

## 3 Methods

### 3.1 Review design

This manuscript was conducted as a single-author narrative review examining the emerging role of engineered probiotic consortia in reducing treatment-associated toxicity and improving chemotherapy tolerance in breast cancer. The review was designed to integrate evidence from microbiome biology, breast cancer treatment, chemotherapy-associated toxicity, conventional probiotics, engineered microbial therapeutics, synthetic biology, immunometabolism, pharmacology, and precision oncology. Because the objective was to provide a broad mechanistic and translational synthesis of an emerging field rather than a quantitative meta-analysis, the review was not designed as a formal systematic review.

### 3.2 Literature search strategy

A structured literature search was undertaken to identify relevant publications addressing the relationship between the gut microbiome, breast cancer, chemotherapy, treatment toxicity, probiotics, engineered probiotics, microbial consortia, and precision supportive oncology. Searches were conducted in PubMed/MEDLINE, Scopus, Web of Science, and Google Scholar, supplemented by reference-list screening of relevant articles and identification of additional studies cited in key reviews.

Searches combined controlled vocabulary and free-text terms related to:

“breast cancer,” “breast neoplasm,” “gut microbiome,” “intestinal microbiota,” “dysbiosis,” “chemotherapy,” “chemotherapy toxicity,” “mucositis,” “diarrhea,” “intestinal barrier,” “probiotics,” “engineered probiotics,” “synthetic biology,” “microbial consortia,” “live biotherapeutics,” “microbiome therapeutics,” “immunometabolism,” “drug metabolism,” “endocrine therapy,” “HER2,” “triple-negative breast cancer,” and “precision oncology.”

Search terms were combined using Boolean operators AND and OR, with database-specific adaptations where necessary. Additional relevant studies were identified through backward and forward citation tracking of highly relevant publications.

### 3.3 Eligibility considerations

Publications were considered relevant when they addressed one or more of the following domains.

1. the relationship between breast cancer and the gut or breast-associated microbiome;
2. chemotherapy-associated microbiome disruption or dysbiosis;
3. microbiome contributions to chemotherapy toxicity or treatment response;
4. conventional probiotic interventions in cancer or chemotherapy;
5. engineered probiotic organisms or defined microbial consortia;
6. synthetic-biology approaches to microbial therapeutics;
7. microbial regulation of epithelial barrier integrity, inflammation, immunity, or metabolism;
8. microbial effects on anticancer drug metabolism or pharmacokinetics;
9. microbiome interactions with endocrine, targeted, or immune-based breast cancer therapy;
10. safety, manufacturing, containment, and regulatory considerations for live microbial therapeutics; and
11. biomarker, multi-omic, computational, or precision-medicine approaches relevant to microbiome-guided supportive oncology.

Both human and preclinical evidence were considered because clinical studies specifically evaluating engineered probiotic consortia in breast cancer remain limited. Human studies were prioritized when making statements concerning clinical efficacy, toxicity reduction, treatment tolerance, or patient outcomes, whereas mechanistic and preclinical studies were used to explain biological plausibility and identify translational hypotheses. Publications were excluded when they were unrelated to breast cancer or cancer-treatment-associated microbiome biology, lacked sufficient relevance to probiotic or microbial therapeutic mechanisms, consisted solely of non-informative commentary, or duplicated information already represented by a more appropriate primary source. Where evidence was derived from cancers other than breast cancer, this was explicitly considered indirect or supportive evidence rather than breast cancer-specific clinical evidence.

### 3.4 Study screening and selection

Titles and abstracts identified through the searches were screened for relevance, followed by assessment of potentially relevant full-text publications. Because this was a single-author narrative review, screening, eligibility assessment, data extraction, interpretation, and quality appraisal were performed by the sole author. No independent second reviewer was available for duplicate screening or adjudication. To reduce selection and interpretation bias, the author used predefined thematic eligibility considerations, reviewed potentially relevant full-text publications, cross-checked key findings against original studies where accessible, and maintained a distinction between preclinical, mechanistic, observational, and interventional clinical evidence. Studies were not treated as equivalent solely because they addressed similar microbiome concepts.

### 3.5 Data extraction and thematic organization

Information was extracted qualitatively according to predefined thematic domains. These included.

- breast cancer subtype and clinical context;
- treatment modality and chemotherapy regimen;
- microbial species or consortium characteristics;
- conventional *versus* engineered probiotic approach;
- microbiome changes associated with treatment;
- proposed mechanisms of toxicity;
- epithelial barrier effects;
- inflammatory and immune pathways;
- microbial metabolites;
- drug–microbiome interactions;
- clinical toxicity outcomes;
- treatment tolerance and treatment delivery;
- antitumor efficacy;
- safety outcomes;
- patient characteristics;
- nutritional and lifestyle factors;
- endocrine or targeted treatment interactions;
- and translational or regulatory considerations.

Where available, information concerning clinical trial registration numbers, study design, sample size, intervention characteristics, treatment setting, and major outcomes was recorded. Individual studies represented in tables were linked to their corresponding references to improve traceability.

### 3.6 Evidence appraisal

Because the review was narrative rather than systematic, formal quantitative risk-of-bias pooling was not undertaken. Nevertheless, evidence was critically appraised according to study design, methodological rigor, biological plausibility, consistency of findings, clinical relevance, and directness to breast cancer.

Evidence was broadly interpreted according to the following hierarchy:

human randomized or controlled clinical evidence → prospective human observational evidence → retrospective or cross-sectional human evidence → translational/*ex vivo* evidence → animal studies → *in vitro* or mechanistic evidence.

Particular caution was applied when findings from other malignancies, non-chemotherapy settings, or conventional probiotics were extrapolated to engineered probiotic consortia in breast cancer. Such evidence was identified as indirect where appropriate.

### 3.7 Breast cancer subtype and treatment stratification

Because breast cancer is biologically heterogeneous, evidence was considered in relation to hormone receptor-positive/HER2-negative, HER2-positive, and triple-negative breast cancer where information was available. The review also considered the potential modifying effects of tumor molecular characteristics, chemotherapy intensity, treatment combinations, menopausal status, obesity, metabolic disease, nutritional status, antibiotic exposure, and other clinically relevant factors. Particular attention was given to anthracycline-, taxane-, cyclophosphamide-, and platinum-containing chemotherapy because these regimens may produce distinct patterns of gastrointestinal injury, systemic inflammation, dysbiosis, and treatment-associated toxicity.

### 3.8 Multi-omic and correlative framework

The review additionally evaluated emerging approaches for integrating microbiome data with other biological and clinical measurements. These included.

- shotgun metagenomics;
- metatranscriptomics;
- microbial metabolomics;
- host inflammatory biomarkers;
- immune-cell profiling;
- intestinal permeability and epithelial injury markers;
- tumor genomics and epigenomics;
- host pharmacogenomics;
- chemotherapy pharmacokinetics and pharmacodynamics;
- nutritional and dietary assessment;
- patient-reported outcomes; and
- longitudinal treatment-response data.

These approaches were considered within a proposed precision framework linking:

tumor subtype and molecular characteristics → treatment exposure → host characteristics → microbiome composition and function → microbial metabolites → immune/inflammatory phenotype → chemotherapy toxicity → treatment delivery → oncological outcome.

### 3.9 Safety and translational assessment

Because engineered probiotic consortia involve live microorganisms and may ultimately be administered to patients receiving immunosuppressive or myelosuppressive treatment, safety considerations were specifically evaluated. These included microbial translocation, bacteremia, fungemia, antimicrobial resistance, horizontal gene transfer, genetic instability, uncontrolled persistence, ecological disruption, drug–microbiome interactions, and unintended immune effects. The translational assessment also considered manufacturing consistency, strain identity and purity, viability, potency, genetic stability, containment, delivery, and regulatory challenges associated with engineered live microbial therapeutics.

### 3.10 Lifestyle, diet, and medication considerations

Recognizing that microbial ecology is strongly influenced by environmental and host factors, the review considered the potential effects of dietary pattern, nutritional status, physical activity, antibiotic exposure, concomitant medications, and other lifestyle factors on microbiome composition and response to microbial interventions. These factors were considered potential modifiers and confounders rather than established determinants of response.

### 3.11 Synthesis of evidence

Findings were synthesized narratively according to major biological and clinical themes rather than statistically pooled. The synthesis focused on five interconnected questions.

1. How does chemotherapy alter the microbiome and intestinal barrier?
2. How might microbial dysbiosis contribute to treatment-associated toxicity?
3. Which microbial functions could potentially be engineered to improve chemotherapy tolerance?
4. How can engineered consortia be personalized according to tumor, treatment, host, and microbiome characteristics?
5. What evidence, safety controls, biomarkers, and clinical trials are required before these interventions can be translated into routine supportive oncology?

Particular emphasis was placed on distinguishing established evidence from mechanistic hypotheses and future opportunities. Claims concerning clinical efficacy were restricted to evidence supported by human studies, whereas proposed applications of engineered consortia were explicitly identified as emerging or investigational when direct clinical evidence was unavailable.

### 3.12 Methodological limitations

The single-author design constitutes an important methodological limitation because independent duplicate screening and adjudication were not possible. In addition, heterogeneity in study populations, probiotic formulations, chemotherapy regimens, microbiome analytical methods, and clinical endpoints limits direct comparison across studies. The rapidly evolving nature of engineered microbial therapeutics also means that some emerging technologies have limited longitudinal safety and efficacy data. Accordingly, this review is intended to provide a critical, mechanistically informed and translational synthesis of an emerging field, rather than to provide pooled estimates of treatment efficacy. Future systematic reviews and meta-analyses will be appropriate when a sufficiently large and homogeneous body of clinical evidence concerning engineered probiotic consortia in breast cancer becomes available.

### 3.13 Breast cancer subtype, tumor biology, and treatment context

Breast cancer is a biologically heterogeneous disease, and the potential role of engineered probiotic consortia should therefore be considered within the context of molecular subtype, tumor biology, treatment regimen, host immune status, and individual toxicity risk. The major clinical subgroups—hormone receptor-positive/HER2-negative, HER2-positive, and triple-negative breast cancer (TNBC)—differ substantially in their molecular characteristics, systemic treatment strategies, immune microenvironment, and patterns of treatment-related toxicity. Consequently, a microbiome-directed supportive intervention developed for breast cancer should not be regarded as a uniform strategy applicable identically across all patients (1). The primary rationale for engineered probiotic consortia is currently not direct targeting of breast cancer mutations or epigenetic alterations. Rather, their proposed role is to modify the host–microbiome interface during systemic anticancer treatment. Tumor molecular characteristics are nevertheless relevant because they influence treatment selection and intensity, which in turn determine the nature and magnitude of chemotherapy-associated gastrointestinal, inflammatory, metabolic, and systemic toxicity. Thus, tumor genomics and epigenomics may serve as important contextual variables for determining which patients are most likely to benefit from microbiome-directed supportive interventions (2).

In hormone receptor-positive/HER2-negative breast cancer, chemotherapy is generally reserved for patients with sufficiently high clinical or molecular risk to justify cytotoxic treatment. In such patients, engineered probiotic strategies could potentially focus on maintaining intestinal barrier integrity, preserving microbial metabolic functions, and reducing treatment-associated gastrointestinal toxicity without interfering with subsequent endocrine or other systemic therapies. In HER2-positive disease, chemotherapy is commonly integrated with HER2-directed treatment, creating a more complex therapeutic environment in which microbiome interventions would need to account for interactions among cytotoxic chemotherapy, targeted therapy, immune signaling, and supportive medications. TNBC is particularly relevant to microbiome research because of its aggressive biology, frequent use of intensive chemotherapy, and substantial interest in immune-mediated mechanisms. In this setting, microbial interventions should be designed to minimize treatment toxicity while avoiding excessive immunosuppression that could theoretically compromise beneficial antitumor immune responses (3, 4). The relationship between breast cancer mutations, epigenetic alterations, and the microbiome remains an emerging area of investigation. Genomic alterations such as BRCA1/BRCA2-associated defects in DNA repair, TP53 abnormalities, and other molecular alterations prim arily determine tumor biology and therapeutic vulnerability rather than serving as direct targets of probiotic therapy. However, these molecular features can influence treatment selection, immune interactions, cellular metabolism, and therapeutic response, potentially creating different host environments in which microbiome-directed interventions may perform differently. Similarly, epigenetic mechanisms involving DNA methylation, histone modification, and non-coding RNAs may interact with metabolic and inflammatory pathways that are also influenced by the microbiome. At present, these relationships should be considered mechanistic hypotheses requiring prospective validation rather than established indications for specific probiotic consortia (5, 6).

The chemotherapy regimen itself may therefore be a more immediate determinant of microbiome-directed supportive-care requirements than tumor genotype alone. Anthracycline-, taxane-, cyclophosphamide-, and platinum-containing regimens can produce different degrees and patterns of gastrointestinal epithelial injury, inflammatory activation, dysbiosis, and systemic toxicity. Combination regimens may impose particularly complex ecological pressures on the intestinal microbiome. Consequently, future clinical studies should stratify patients according to breast cancer subtype, chemotherapy regimen, treatment intensity, baseline microbiome composition, antibiotic exposure, nutritional status, immune competence, and previous treatment-related toxicity (7). An additional consideration is that microbiome manipulation should not be evaluated solely according to its capacity to reduce gastrointestinal symptoms. The microbiome can influence immune activation, microbial metabolite production, drug metabolism, and systemic inflammatory pathways, potentially affecting both toxicity and therapeutic efficacy. A consortium that improves intestinal tolerance but alters anticancer drug exposure or suppresses beneficial immune responses would not represent a successful precision-supportive intervention. Therefore, future trials should simultaneously evaluate toxicity, chemotherapy dose intensity, pharmacokinetics, treatment response, and oncological outcomes (8). The proposed target population should consequently be defined as selected breast cancer patients receiving systemic chemotherapy who have substantial risk of, or established, treatment-associated gastrointestinal and microbiome-related toxicity. This may include patients experiencing diarrhea, mucosal injury, intestinal barrier dysfunction, dysbiosis, gastrointestinal inflammation, nutritional disturbance, or toxicity sufficiently severe to cause treatment interruption or dose modification. The approach should not currently be generalized to all breast cancer treatments, total-body irradiation, targeted therapy, immunosuppressive therapy, or immunotherapy without treatment-specific evidence. In patients with profound neutropenia, severe mucosal barrier disruption, active infection, or other major risk factors for microbial translocation, administration of live microbial therapeutics requires particular caution.

### 3.14 Target population and scope of toxicity reduction

The proposed use of engineered probiotic consortia is intended primarily for selected breast cancer patients receiving systemic cytotoxic chemotherapy who are at substantial risk of, or have developed, treatment-associated gastrointestinal and microbiome-related toxicity. The objective is not to provide a universal intervention for all patients with breast cancer, but to develop a precision supportive-care strategy tailored to the treatment regimen, toxicity phenotype, baseline microbiome, host characteristics, and overall clinical risk profile. This distinction is important because breast cancer encompasses biologically and therapeutically heterogeneous disease subtypes, and the magnitude and pattern of treatment toxicity vary considerably between patients. The principal toxicities of interest include chemotherapy-associated diarrhea, intestinal mucosal injury, gastrointestinal inflammation, altered intestinal permeability, dysbiosis, abdominal symptoms, nutritional disturbances, and systemic inflammatory consequences of intestinal barrier disruption. These complications may impair quality of life, reduce nutritional and functional status, necessitate additional supportive medications, increase hospitalization, result in dose reductions or treatment delays, and ultimately compromise the ability to deliver the planned anticancer regimen. Accordingly, the clinically meaningful objective of an engineered probiotic intervention is not simply improvement in microbial diversity or reduction of an isolated gastrointestinal symptom, but improved tolerance and completion of effective anticancer therapy without compromising tumor control.

The potential target population may include patients receiving anthracycline-, taxane-, cyclophosphamide-, or platinum-containing chemotherapy, either individually or as components of combination regimens, when clinically significant gastrointestinal or microbiome-associated toxicity is anticipated or observed. Patients with previous severe chemotherapy-associated gastrointestinal toxicity may represent an especially relevant population for prospective investigation. Similarly, patients demonstrating marked baseline dysbiosis, reduced microbial functional capacity, altered microbial metabolite profiles, or other biomarkers associated with increased toxicity risk may eventually be candidates for biomarker-guided microbial intervention. However, these characteristics should currently be regarded as investigational selection criteria rather than established clinical indications. The scope should not be interpreted as extending automatically to total-body irradiation (TBI), radiotherapy, targeted therapies, endocrine therapy, immunotherapy, or immunosuppressive treatments. These modalities may produce distinct patterns of tissue injury, immune modulation, and microbiome alteration, and therefore require treatment-specific investigation. Engineered probiotic consortia could potentially have applications in some of these settings, particularly where gastrointestinal barrier dysfunction or microbiome disruption is clinically relevant, but evidence from cytotoxic chemotherapy should not be extrapolated without appropriate clinical validation.

The concept is also distinct from the treatment of all forms of chemotherapy toxicity. Hematological toxicity, cardiotoxicity, nephrotoxicity, hepatotoxicity, neurotoxicity, and other organ-specific adverse effects may involve mechanisms that cannot be adequately addressed through microbiome manipulation alone. Although the microbiome may indirectly influence systemic metabolism and inflammation, the current therapeutic rationale is strongest for toxicities involving the gastrointestinal tract, intestinal barrier, microbial ecology, and related inflammatory pathways. Patient safety represents an essential component of population selection. Individuals receiving intensive chemotherapy may develop neutropenia, lymphopenia, mucosal barrier injury, central venous access, or other conditions that increase susceptibility to infection. Administration of live microbial therapeutics in patients with profound neutropenia, severe intestinal barrier disruption, active systemic infection, uncontrolled sepsis, or other major risk factors for microbial translocation therefore requires particular caution. Early clinical trials should use stringent eligibility criteria, microbiological surveillance, and predefined stopping rules for infectious or unexpected systemic complications. The proposed population should also be stratified according to breast cancer molecular subtype, chemotherapy regimen, treatment intensity, baseline microbiome composition, antibiotic exposure, nutritional status, immune phenotype, comorbidities, and previous treatment-related toxicity. Such stratification is necessary because two patients receiving nominally similar chemotherapy may have markedly different microbial ecosystems and toxicity trajectories. Future predictive models could combine clinical variables with metagenomic, metabolomic, inflammatory, and pharmacological data to identify patients most likely to benefit.

The goal of toxicity reduction should not be separated from oncological efficacy. Microbiome manipulation can potentially influence immune responses and drug metabolism, creating the possibility that an intervention designed to improve tolerability could inadvertently modify chemotherapy pharmacokinetics or antitumor activity. Consequently, clinical trials should evaluate not only gastrointestinal toxicity but also chemotherapy dose intensity, treatment completion, pharmacokinetic parameters, objective tumor response, progression-free survival, and overall survival. The appropriate conceptual framework is therefore selective, biomarker-informed supportive intervention rather than generalized probiotic supplementation. Engineered probiotic consortia should be developed for clearly defined toxicity phenotypes and administered only when the anticipated benefits outweigh potential infectious, ecological, pharmacological, and immunological risks. Future precision-supportive strategies may ultimately follow a pathway of baseline risk assessment → microbiome and metabolomic profiling → chemotherapy-specific toxicity prediction → consortium selection → longitudinal monitoring → treatment adaptation. In this context, the principal target population can be summarized as breast cancer patients receiving systemic cytotoxic chemotherapy who are at elevated risk for clinically meaningful gastrointestinal and microbiome-associated toxicity and who can safely receive a rigorously characterized microbial therapeutic. This focused definition provides a more clinically realistic framework for evaluating engineered probiotic consortia while avoiding unsupported extrapolation to unrelated treatment modalities or toxicities.

### 3.15 The gut microbiome and chemotherapy toxicity

The gut microbiome has emerged as a critical determinant of chemotherapy response and toxicity in cancer patients. Comprising trillions of microorganisms, including bacteria, fungi, viruses, and archaea, the intestinal microbiota functions as a complex metabolic and immunological organ that influences host physiology through regulation of nutrient metabolism, immune homeostasis, epithelial barrier integrity, and inflammatory signaling. In breast cancer patients, growing evidence indicates that interactions between chemotherapeutic agents and the gut microbiome significantly affect both treatment efficacy and the development of adverse events. Consequently, the microbiome is increasingly recognized as a key mediator of chemotherapy tolerance and an attractive target for supportive therapeutic interventions (10). Chemotherapy-induced toxicity is traditionally attributed to the direct cytotoxic effects of anticancer drugs on rapidly dividing normal tissues, particularly the gastrointestinal epithelium, bone marrow, and mucosal surfaces. However, recent studies have demonstrated that many toxicities arise not only from direct tissue injury but also from treatment-induced alterations in microbial communities. Cytotoxic agents can profoundly disrupt gut microbial composition, resulting in dysbiosis characterized by reduced microbial diversity, depletion of beneficial commensal bacteria, and expansion of opportunistic pathogens. These changes may exacerbate inflammation, impair epithelial repair mechanisms, and increase susceptibility to treatment-related complications (11).

Several commonly used breast cancer chemotherapeutic agents, including anthracyclines, cyclophosphamide, taxanes, fluoropyrimidines, and platinum compounds, have been shown to alter intestinal microbial ecosystems. Chemotherapy often reduces populations of beneficial bacteria such as *Lactobacillus*, *Bifidobacterium*, *Faecalibacterium prausnitzii*, *Roseburia*, and *Akkermansia muciniphila*. These organisms are essential for maintaining intestinal barrier function, producing short-chain fatty acids (SCFAs), suppressing inflammation, and regulating immune responses. Their depletion may compromise mucosal integrity and contribute to increased intestinal permeability, commonly referred to as “leaky gut.” This disruption allows microbial products such as lipopolysaccharides (LPS) and other pathogen-associated molecular patterns to enter systemic circulation, triggering inflammatory cascades that amplify chemotherapy-induced tissue damage (12, 13). One of the most clinically significant manifestations of chemotherapy-associated dysbiosis is gastrointestinal mucositis. Mucositis results from a complex interplay between direct epithelial injury, oxidative stress, inflammatory cytokine production, and microbial alterations. Damage to the intestinal lining facilitates bacterial translocation and activation of innate immune pathways, leading to increased production of pro-inflammatory mediators such as tumor necrosis factor-alpha (TNF-α), interleukin-1β (IL-1β), and interleukin-6 (IL-6). These cytokines further perpetuate mucosal injury, creating a self-reinforcing cycle of inflammation and tissue destruction. Patients experiencing severe mucositis often develop pain, diarrhea, malnutrition, dehydration, and increased risk of infection, frequently necessitating chemotherapy dose reductions or treatment delays (14, 15).

Short-chain fatty acids, particularly butyrate, acetate, and propionate, represent a crucial link between the gut microbiome and chemotherapy toxicity. These microbial metabolites are generated through fermentation of dietary fibers and play essential roles in maintaining epithelial health and immune regulation. Butyrate serves as the primary energy source for colonocytes, promotes tight junction integrity, enhances mucosal repair, and exerts potent anti-inflammatory effects. Chemotherapy-induced depletion of SCFA-producing bacteria reduces the availability of these protective metabolites, thereby increasing susceptibility to mucosal injury and systemic inflammation. Reduced SCFA production has been associated with greater gastrointestinal toxicity and impaired recovery following chemotherapy (16). The microbiome also influences chemotherapy toxicity through modulation of oxidative stress pathways. Many anticancer drugs generate reactive oxygen species (ROS) that contribute to both tumor cell killing and collateral damage to healthy tissues. Beneficial commensal bacteria produce antioxidant compounds and stimulate host antioxidant defense systems that help neutralize excessive ROS accumulation. Dysbiosis may impair these protective mechanisms, resulting in heightened oxidative damage to gastrointestinal tissues, liver cells, and other organs vulnerable to chemotherapy-induced injury (17). Immune dysregulation represents another important mechanism linking microbiome alterations to treatment-related adverse effects. The gut microbiota plays a central role in educating and regulating the immune system through interactions with intestinal immune cells. Beneficial microbial species promote the development of regulatory T cells and maintain balanced immune responses, whereas dysbiosis often favors pro-inflammatory immune activation. Chemotherapy-induced microbial disturbances may therefore contribute to excessive cytokine release, systemic inflammation, and impaired immune recovery. Such effects may increase the risk of infections, delay hematopoietic regeneration, and worsen overall treatment tolerance (18).

Emerging evidence further suggests that the gut microbiome influences chemotherapy-induced myelosuppression, one of the most common dose-limiting toxicities in breast cancer treatment. Microbial metabolites regulate hematopoietic stem cell function, bone marrow homeostasis, and leukocyte production. Alterations in microbial composition may disrupt these regulatory pathways, potentially contributing to neutropenia, anemia, and thrombocytopenia. Animal studies have demonstrated that restoration of healthy microbial communities can improve hematopoietic recovery and enhance resistance to chemotherapy-induced bone marrow suppression (19). In addition to gastrointestinal and hematological toxicities, microbiome-mediated mechanisms may contribute to systemic adverse effects such as fatigue, neurotoxicity, hepatotoxicity, and metabolic dysfunction. The gut-brain axis provides a bidirectional communication network linking intestinal microorganisms with the central nervous system through neural, endocrine, and immune pathways. Chemotherapy-induced dysbiosis has been associated with altered neurotransmitter production, increased neuroinflammation, and cognitive impairment, suggesting a potential role in cancer-related fatigue and chemotherapy-associated cognitive dysfunction. Similarly, microbial metabolites influence hepatic detoxification pathways and may affect susceptibility to drug-induced liver injury (20).

**FIGURE 1:**
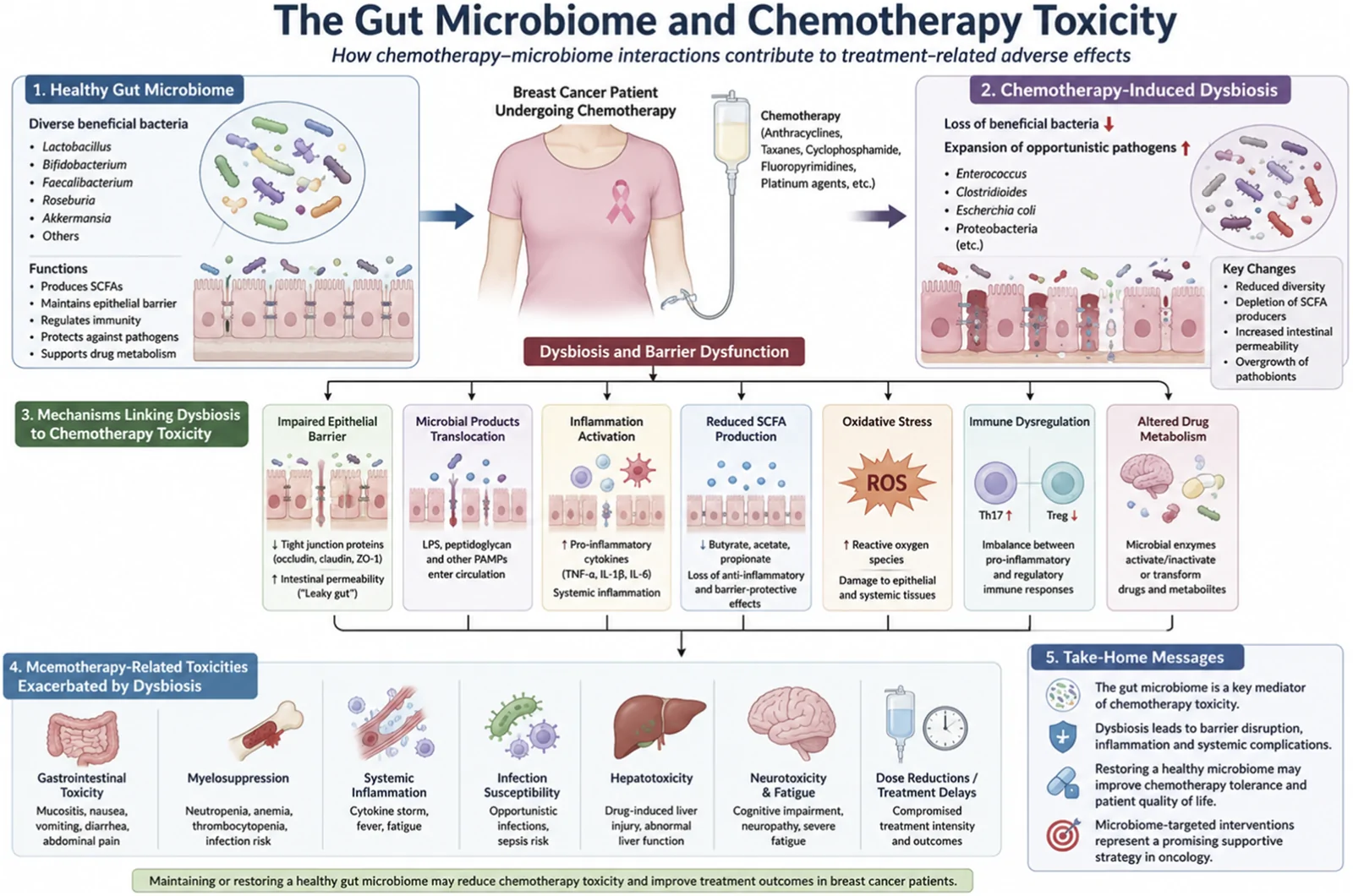
The gut microbiome and chemotherapy toxicity.

The relationship between chemotherapy and the gut microbiome is bidirectional. While chemotherapy alters microbial composition, the microbiome itself influences drug metabolism, bioavailability, and toxicity. Certain bacterial species possess enzymatic activities capable of activating, inactivating, or transforming chemotherapeutic agents and their metabolites. These microbial biotransformation processes may modify treatment toxicity profiles and contribute to interindividual variability in adverse event severity. Understanding these complex host-microbe-drug interactions is therefore essential for developing personalized approaches to toxicity management (21). Advances in metagenomics, metabolomics, and systems biology have greatly expanded understanding of the microbiome’s role in chemotherapy toxicity. These technologies have identified specific microbial signatures associated with favorable or unfavorable treatment outcomes, opening opportunities for microbiome-guided supportive care strategies. Interventions aimed at preserving microbial diversity, restoring beneficial bacterial populations, and enhancing production of protective metabolites are increasingly being investigated as methods to improve chemotherapy tolerance in breast cancer patients (Figure 1) (22).

## 4 Pathophysiological basis of chemotherapy-induced toxicity and microbiome dysbiosis

Chemotherapy-induced toxicity in breast cancer patients is a multifactorial process, involving direct cytotoxic effects on rapidly dividing cells, disruption of tissue homeostasis, immune dysregulation, and systemic inflammatory responses. While cytotoxic agents effectively target tumor cells, they simultaneously affect healthy proliferative tissues, particularly the gastrointestinal epithelium, bone marrow, and mucosal immune cells. This dual impact initiates a cascade of pathological events that underpin both local and systemic toxicities, including mucositis, diarrhea, myelosuppression, fatigue, and metabolic disturbances (10, 11). At the mucosal level, chemotherapy induces apoptosis in basal epithelial cells, leading to thinning of the mucosal lining and disruption of tight junctions. The loss of epithelial integrity increases permeability, allowing luminal bacteria and microbial products to translocate into the submucosa and systemic circulation. This breach triggers activation of innate immune responses, including recruitment of neutrophils and macrophages, and amplification of pro-inflammatory signaling pathways such as NF-κB, TNF-α, IL-1β, and IL-6. Oxidative stress generated by chemotherapy further exacerbates epithelial injury, contributing to a feed-forward loop of inflammation and tissue damage (12, 13).

Concomitantly, chemotherapy profoundly alters the gut microbiome, producing a state of dysbiosis that amplifies mucosal vulnerability and systemic toxicity. Loss of microbial diversity reduces ecological resilience and functional redundancy, while depletion of beneficial commensals such as *Lactobacillus*, Bifidobacterium, Faecalibacterium prausnitzii, and Akkermansia muciniphila impairs short-chain fatty acid (SCFA) production, mucin turnover, and anti-inflammatory signaling. The expansion of pathobionts, including *Enterococcus* and Enterobacteriaceae, further destabilizes the ecosystem, promoting endotoxin release, toll-like receptor activation, and enhanced inflammatory responses. These microbiome alterations exacerbate mucosal injury, delay epithelial repair, and increase the risk of systemic infections, particularly in immunocompromised patients (14–25). Functional consequences of dysbiosis extend beyond compositional changes. Chemotherapy-induced microbial shifts impair critical metabolic processes, including SCFA biosynthesis, bile acid transformation, and amino acid metabolism, all of which are essential for epithelial regeneration and immunomodulation. The resulting metabolic deficits contribute to prolonged mucositis, increased oxidative stress, and systemic inflammatory burden. Additionally, microbiome perturbations can influence drug metabolism, potentially altering chemotherapeutic pharmacokinetics and pharmacodynamics, which may further impact treatment efficacy and toxicity profiles (16, 17). The interplay between epithelial injury, immune activation, and microbiome disruption establishes a vicious cycle: epithelial damage promotes dysbiosis, dysbiosis exacerbates inflammation, and inflammation further injures epithelial tissues. This cycle underlies not only gastrointestinal toxicity but also systemic side effects, including fatigue, anorexia, and myelosuppression, which collectively compromise chemotherapy tolerance and patient quality of life (Table 1) (18).

**TABLE 1:** Pathophysiological basis of chemotherapy-induced toxicity and microbiome dysbiosis.

Pathophysiological mechanism	Chemotherapy-induced changes	Microbiome alterations	Biological consequences	Clinical manifestations
Disruption of intestinal epithelial barrier	Direct damage to rapidly dividing intestinal epithelial cells; loss of tight junction integrity	Depletion of beneficial bacteria (*Lactobacillus*, *Bifidobacterium*, *Akkermansia*) that support mucosal health	Increased intestinal permeability (“leaky gut”), bacterial translocation, impaired mucosal repair	Mucositis, abdominal pain, diarrhea, malabsorption, dehydration
Loss of microbial diversity (dysbiosis)	Cytotoxic stress alters gut microbial ecology	Reduced microbial richness and depletion of commensal species; expansion of opportunistic pathogens	Ecosystem instability, impaired colonization resistance, altered microbial metabolism	Increased susceptibility to infections and gastrointestinal complications
Microbial product translocation	Barrier disruption permits passage of microbial components into circulation	Increased release of lipopolysaccharides (LPS), peptidoglycans, and microbial toxins	Activation of toll-like receptors (TLRs) and innate immune responses	Systemic inflammation, fever, fatigue, cytokine-mediated toxicity
Inflammatory cytokine activation	Tissue injury stimulates inflammatory signaling pathways	Dysbiosis promotes excessive pro-inflammatory microbial interactions	Increased TNF-α, IL-1β, IL-6, NF-κB activation, and inflammasome signaling	Mucositis, cachexia, fatigue, treatment intolerance
Reduced short-chain fatty acid (SCFA) production	Loss of SCFA-producing bacteria following chemotherapy exposure	Decreased abundance of *Faecalibacterium*, *Roseburia*, and related butyrate-producing species	Reduced epithelial regeneration, impaired immune regulation, increased inflammation	Persistent gastrointestinal toxicity and delayed mucosal recovery
Oxidative stress amplification	Generation of reactive oxygen species (ROS) by chemotherapeutic agents	Reduction of antioxidant-producing microbial populations	Increased oxidative tissue damage, mitochondrial dysfunction, cellular apoptosis	Mucositis, hepatotoxicity, fatigue, organ dysfunction
Immune dysregulation	Altered immune cell populations and cytokine networks	Disruption of microbiota-mediated immune homeostasis	Reduced regulatory T-cell activity and enhanced pro-inflammatory responses	Increased inflammatory complications and infection risk
Altered drug metabolism	Chemotherapy affects microbial enzymatic activity	Changes in microbial drug-metabolizing enzymes and metabolic pathways	Altered drug bioavailability, toxicity, and pharmacokinetics	Variable treatment response and toxicity severity among patients
Bone marrow and hematopoietic dysfunction	Direct suppression of hematopoietic stem cells by chemotherapy	Reduced production of microbiota-derived hematopoietic-supporting metabolites	Impaired leukocyte, erythrocyte, and platelet recovery	Neutropenia, anemia, thrombocytopenia, infection susceptibility
Gut-brain axis disturbance	Chemotherapy-induced neuroinflammation and metabolic changes	Altered production of microbial neurotransmitters and neuroactive metabolites	Increased neuroinflammation, altered neural signaling, impaired cognition	Chemotherapy-related cognitive impairment (“chemobrain”), fatigue, mood disturbances
Hepatic-microbial axis dysfunction	Hepatotoxic effects of chemotherapy alter host metabolism	Dysbiosis affects bile acid metabolism and hepatic detoxification pathways	Increased liver inflammation and metabolic stress	Elevated liver enzymes, hepatotoxicity, impaired drug clearance
Opportunistic pathogen expansion	Immune suppression and ecological disruption favor pathogen overgrowth	Increased abundance of *Enterococcus*, *Escherichia coli*, *Clostridioides*, and proteobacteria	Enhanced pathogen colonization and toxin production	Gastrointestinal infections, bacteremia, sepsis, prolonged hospitalization

Abbreviations: IL-1β = Interleukin-1, beta; IL-6, Interleukin-6; LPS, lipopolysaccharide; NF-κB, Nuclear Factor kappa B; ROS, reactive oxygen species; SCFA, Short-Chain Fatty Acid; TLR, Toll-Like Receptor; TNF-α, Tumor Necrosis Factor-alpha.

## 5 Mechanistic rationale for engineering probiotic consortia

The concept of engineering probiotic consortia to support breast cancer patients undergoing chemotherapy arises from an appreciation of the complex, multidimensional roles that the gut microbiome plays in maintaining gastrointestinal and systemic homeostasis. Chemotherapy disrupts the microbial ecosystem, depleting beneficial commensals, expanding pathobionts, and impairing critical metabolic functions. Single-strain probiotics, while beneficial in certain contexts, often lack the functional breadth and ecological resilience required to fully restore microbial balance or mitigate the multifactorial toxicity induced by cytotoxic therapy. In this context, rationally designed probiotic consortia offer a compelling mechanistic strategy by combining multiple microbial strains with complementary functions that collectively reinforce epithelial integrity, modulate immune responses, and restore metabolic homeostasis (19, 20).

Probiotic consortia can restore the production of short-chain fatty acids (SCFAs), particularly butyrate, propionate, and acetate, which serve as critical energy sources for colonocytes and play a central role in maintaining tight junction integrity and epithelial barrier function. By replenishing SCFA-producing taxa, such consortia help to counteract chemotherapy-induced epithelial apoptosis and support mucosal regeneration. Beyond their metabolic contributions, engineered consortia can also influence immune modulation. Certain strains stimulate regulatory T cell activity, enhance anti-inflammatory cytokine signaling, and suppress pro-inflammatory pathways such as NF-κB, thereby attenuating the excessive immune activation that contributes to mucositis and systemic toxicity (21, 22). In addition to metabolic and immunomodulatory functions, probiotic consortia provide competitive ecological advantages, limiting the expansion of pathobionts that can exacerbate mucosal injury. Strains within the consortium can occupy distinct niches within the gut ecosystem, produce antimicrobial compounds, and maintain colonization stability, effectively suppressing the proliferation of opportunistic bacteria such as *Enterococcus* and Enterobacteriaceae. This ecological strategy reduces microbial-driven inflammation and prevents the amplification of epithelial injury through bacterial translocation and endotoxin-mediated signaling (23).

The complementary nature of engineered consortia extends to the restoration of essential microbial metabolites beyond SCFAs. By collectively producing vitamins, amino acids, and antioxidant molecules, these consortia support systemic resilience, counteract oxidative stress, and enhance tissue repair processes disrupted by chemotherapy. Rational selection of strains ensures functional redundancy, so that if one microbial population is diminished by cytotoxic therapy, others can maintain critical metabolic and immunological functions (24). Moreover, advances in synthetic biology and microbial engineering allow for precision design of probiotic consortia. Strains can be engineered to sense inflammatory or oxidative stress signals and respond by producing protective molecules, including anti-inflammatory cytokines or growth factors, in a temporally and spatially controlled manner. Encapsulation and targeted delivery technologies further enhance survival through gastric transit and colonization of specific gut niches, maximizing therapeutic efficacy (Table 2) (25).

**TABLE 2:** Mechanistic rationale for engineering probiotic consortia to improve chemotherapy tolerance in breast cancer patients.

Therapeutic mechanism	Engineering strategy	Target biological pathway	Expected effect on chemotherapy toxicity	Potential clinical benefit
Restoration of microbial diversity	Introduction of complementary probiotic strains with synergistic functions	Microbial ecosystem stability and colonization resistance	Reversal of chemotherapy-induced dysbiosis	Improved gut health and treatment tolerance
Enhancement of intestinal barrier integrity	Engineering strains to produce mucins, tight-junction regulators, and epithelial growth factors	Tight junction proteins (occludin, claudins, ZO-1) and mucosal repair pathways	Reduced intestinal permeability and bacterial translocation	Lower incidence of mucositis and diarrhea
Short-chain fatty acid (SCFA) production	Enrichment of butyrate-, acetate-, and propionate-producing bacteria	Epithelial metabolism, anti-inflammatory signaling, and immune regulation	Enhanced mucosal healing and reduced inflammation	Improved gastrointestinal recovery during chemotherapy
Anti-inflammatory cytokine modulation	Engineering strains to induce IL-10 and suppress pro-inflammatory mediators	NF-κB, TNF-α, IL-1β, IL-6, and inflammasome pathways	Attenuation of excessive inflammatory responses	Reduced systemic toxicity and fatigue
Detoxification of chemotherapy metabolites	Incorporation of strains expressing detoxifying enzymes and antioxidant pathways	Drug metabolite neutralization and oxidative stress control	Reduced accumulation of toxic by-products	Decreased gastrointestinal and hepatic toxicity
Reactive oxygen species (ROS) scavenging	Engineering microbial antioxidant production systems	Oxidative stress and mitochondrial injury pathways	Reduction of oxidative tissue damage	Protection against mucosal and organ injury
Immune homeostasis restoration	Promotion of regulatory immune responses through microbial signaling molecules	Regulatory T-cell induction and immune tolerance pathways	Balanced immune activation during treatment	Reduced inflammatory complications and infection risk
Suppression of opportunistic pathogens	Engineering bacteriocin-producing and pathogen-excluding strains	Competitive exclusion and antimicrobial peptide production	Prevention of pathogenic bacterial overgrowth	Lower rates of gastrointestinal infections and sepsis
Support of hematopoiesis	Engineering microbes to produce hematopoietic-supportive metabolites and vitamins	Bone marrow recovery and stem cell function pathways	Enhanced recovery from myelosuppression	Reduced neutropenia, anemia, and thrombocytopenia
Modulation of drug metabolism	Designing strains with controlled enzymatic drug-processing capabilities	Microbial pharmacokinetic and pharmacodynamic pathways	More predictable drug metabolism and reduced toxicity variability	Improved chemotherapy safety and personalization
Gut–Brain axis regulation	Production of neuroactive metabolites and neurotransmitter precursors	Neuroimmune and neuroendocrine signaling pathways	Reduced neuroinflammation and cognitive dysfunction	Improved fatigue, mood, and quality of life
Bile acid and hepatic metabolism regulation	Engineering strains involved in bile acid transformation and metabolic homeostasis	Liver-gut axis and detoxification pathways	Reduced hepatic stress and improved drug clearance	Lower risk of hepatotoxicity
Biosensing and responsive therapeutics	Incorporation of genetic circuits activated by inflammation or tissue injury	Real-time detection of pathological signals	Targeted release of therapeutic molecules when needed	Precision toxicity management
Targeted delivery of therapeutic molecules	Engineering probiotics as live biotherapeutic delivery systems	Localized release of anti-inflammatory proteins, peptides, and metabolites	Enhanced efficacy with minimal systemic exposure	Improved safety and therapeutic specificity
Personalized microbiome reprogramming	AI-guided design of patient-specific microbial consortia	Precision microbiome modulation based on individual profiles	Tailored toxicity prevention strategies	Optimized supportive care and treatment outcomes

Abbreviations: AI, artificial intelligence; IL-1β = Interleukin-1, beta; IL-6, Interleukin-6; IL-10, Interleukin-10; NF-κB, Nuclear Factor kappa B; ROS, reactive oxygen species; SCFA, Short-Chain Fatty Acid; TNF-α, Tumor Necrosis Factor-alpha; ZO-1, Zonula Occludens-1.

### 5.1 Preclinical and clinical evidence

A growing body of preclinical and clinical research supports the use of probiotic consortia to mitigate chemotherapy-induced toxicity and improve treatment tolerance. In preclinical studies, murine models of chemotherapy have demonstrated that multi-strain probiotic formulations can substantially reduce gastrointestinal mucosal injury, decrease inflammatory cytokine expression, and enhance epithelial regeneration. For example, combinations of *Lactobacillus* rhamnosus, Bifidobacterium longum, and Faecalibacterium prausnitzii have been shown to restore short-chain fatty acid production, preserve mucin layers, and maintain epithelial tight junction integrity following administration of cyclophosphamide or 5-fluorouracil. These interventions not only protect the intestinal barrier but also reduce systemic inflammatory markers and oxidative stress, highlighting the mechanistic interplay between microbial function and host resilience (26, 27).

**FIGURE 2:**
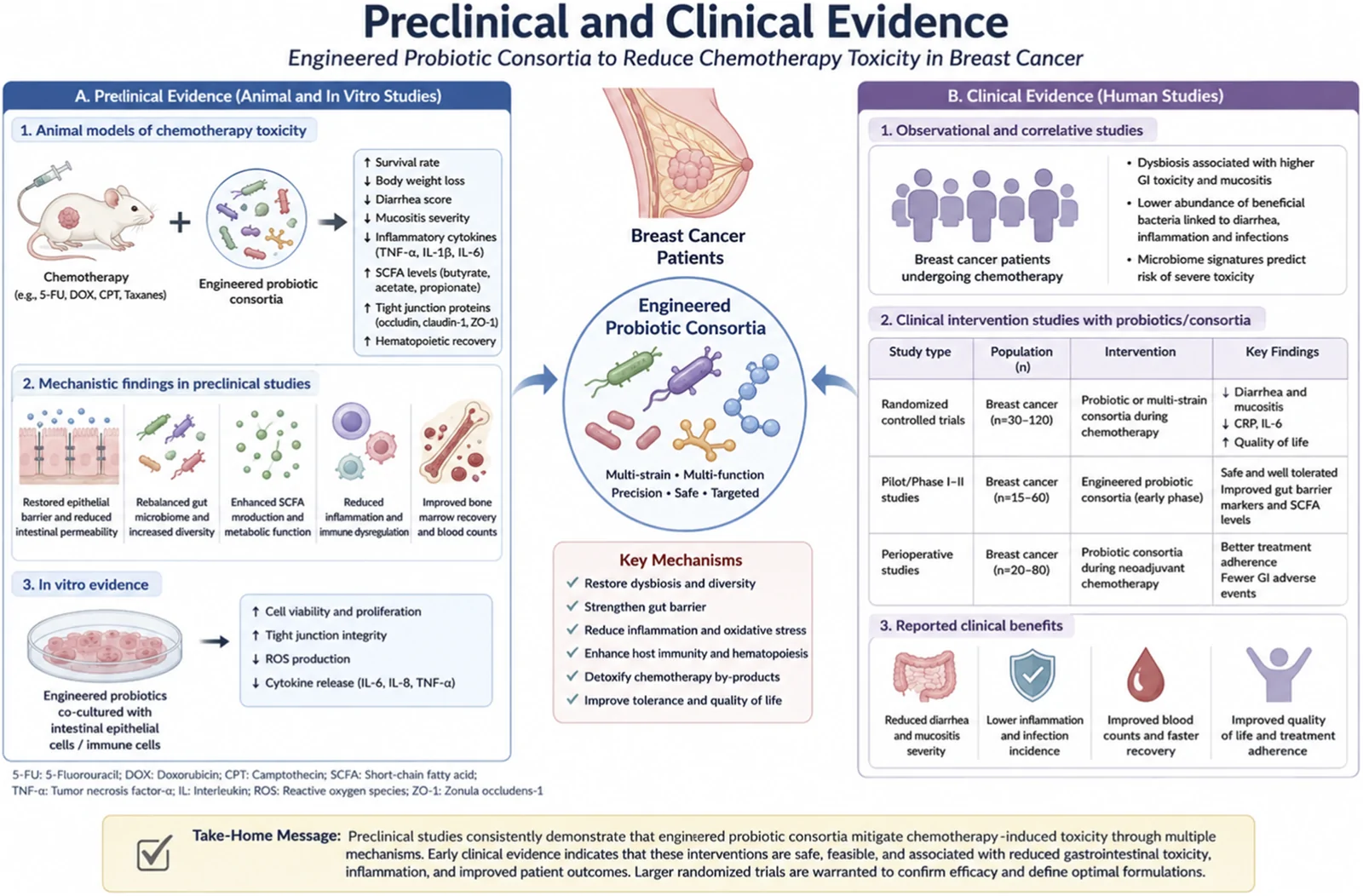
Preclinical and clinical evidence.

Engineered consortia with defined functional properties have further expanded the preclinical evidence base. Microbes designed to secrete anti-inflammatory cytokines, antioxidant molecules, or epithelial growth factors demonstrate enhanced protection against chemotherapy-induced mucosal damage compared with unmodified strains. These studies underscore the principle that rational design—selecting strains based on complementary metabolic and immunomodulatory functions—can amplify therapeutic efficacy, offering a more robust and reproducible response than conventional single-strain probiotics (28, 29). Clinical studies, while more limited, support the feasibility, safety, and efficacy of multi-strain probiotics in oncology patients. Randomized controlled trials in breast and gastrointestinal cancer populations have shown that multi-strain formulations can reduce the incidence and severity of diarrhea, mucositis, and other gastrointestinal toxicities during chemotherapy. Synbiotic approaches, which combine probiotics with prebiotics to enhance microbial growth and metabolic activity, have demonstrated additional benefits, including faster mucosal recovery and improved patient-reported quality of life. Importantly, these interventions appear to be well tolerated, even in patients receiving intensive cytotoxic regimens, with minimal adverse events reported (30, 31). Emerging clinical trials are increasingly exploring the application of rationally engineered consortia tailored to individual patient profiles. Multi-omics approaches, integrating microbiome composition, metabolomics, and immune profiling, allow researchers to identify patients at higher risk of dysbiosis-driven toxicity and to select consortia that complement their microbial deficits. Early-phase studies suggest that personalized consortia can enhance microbial resilience, reduce inflammatory responses, and improve chemotherapy adherence, although larger, breast cancer-specific trials are needed to validate these findings (Figure 2) (32, 33).

### 5.2 Technological advances in probiotic engineering

The development of engineered probiotic consortia has been propelled by advances in synthetic biology, systems microbiology, and precision delivery technologies. These innovations enable the rational design of multi-strain microbial communities with defined functional capacities, ecological stability, and therapeutic specificity, creating new possibilities for mitigating chemotherapy-induced toxicity in breast cancer patients (34). Synthetic biology allows for the precise manipulation of microbial genomes to confer desired functional traits. Strains can be engineered to produce anti-inflammatory cytokines, antioxidants, or growth factors in response to local environmental cues such as oxidative stress or inflammatory signaling. For example, Lactococcus lactis and other commensals have been modified to secrete interleukin-10 or keratinocyte growth factor, demonstrating protective effects on epithelial tissues in preclinical models of chemotherapy-induced mucositis. Such engineered strains can act as responsive, biologically active therapeutics that dynamically adapt to host conditions, providing targeted and temporally controlled interventions (35, 36).

Beyond individual strain engineering, systems biology approaches inform the design of multi-strain consortia with complementary metabolic and immunomodulatory functions. Computational modeling can predict ecological compatibility, niche occupancy, and synergistic interactions, ensuring that strains collectively provide functional redundancy and resilience. This allows the consortia to maintain therapeutic efficacy even if certain populations are diminished by chemotherapy-induced perturbations. Strains can be selected to restore short-chain fatty acid production, produce essential vitamins and amino acids, modulate immune responses, and competitively inhibit pathogenic bacteria, creating a holistic strategy for supporting mucosal and systemic health (37, 38). Targeted delivery and formulation technologies have further enhanced the clinical potential of probiotic consortia. Microencapsulation, biofilm carriers, and pH-sensitive coatings protect microbes during gastric transit, ensuring effective colonization in the colon or small intestine. Controlled-release formulations enable sustained delivery of metabolically active microbes, enhancing persistence and functional output. In addition, synbiotic approaches—combining probiotics with prebiotics—optimize colonization and metabolic activity, allowing for tailored interventions that respond to individual microbial deficits (39). High-throughput sequencing, metagenomics, and metabolomics facilitate the identification of patient-specific microbial imbalances and functional deficits, providing a foundation for precision consortia design. By integrating these multi-omics data with patient immune profiles and chemotherapy regimens, it is possible to select or engineer consortia that complement endogenous microbiota, restore critical metabolic pathways, and modulate host responses. This precision-guided approach transforms probiotic therapy from a generalized supportive measure into a tailored intervention capable of enhancing chemotherapy tolerance and reducing toxicity (40).

### 5.3 Microbiome–endocrine therapy interface in breast cancer

The interaction between the intestinal microbiome and endocrine therapy represents an important but comparatively underdeveloped component of microbiome-informed precision oncology, particularly for estrogen receptor-positive (ER-positive) breast cancer. Unlike cytotoxic chemotherapy, endocrine therapy is designed to suppress estrogen signaling or inhibit estrogen-receptor activity, and its therapeutic success depends on sustained suppression of hormone-driven tumor growth. The gut microbiome may influence this therapeutic environment through estrogen metabolism, enterohepatic circulation, microbial metabolite production, systemic inflammation, and potentially drug metabolism. However, evidence directly demonstrating that engineered probiotic consortia improve endocrine therapy efficacy remains limited, and these concepts should currently be regarded as investigational (41). The intestinal microbial community contains genes encoding enzymes involved in estrogen metabolism and is often collectively described as the estrobolome. Microbial β-glucuronidase activity can deconjugate estrogen metabolites that have undergone hepatic glucuronidation, potentially facilitating their reabsorption through the enterohepatic circulation. Consequently, changes in microbial composition and enzymatic activity may influence the systemic availability and metabolism of estrogen-related compounds. This mechanism is particularly relevant to ER-positive breast cancer because estrogen availability is a major determinant of tumor-cell signaling. However, the relationship is complex. Alterations in the estrobolome do not necessarily translate into clinically meaningful changes in circulating estrogen concentrations, and microbiome composition alone cannot be assumed to predict endocrine treatment response (42).

Tamoxifen is a selective estrogen-receptor modulator whose therapeutic activity depends substantially on hepatic conversion to active metabolites, particularly endoxifen. Although hepatic cytochrome P450 enzymes remain central to this process, the broader intestinal microbiome may influence drug metabolism indirectly through effects on enterohepatic circulation, inflammation, nutritional status, and host metabolic pathways. Microbiome composition may also influence treatment-associated symptoms and adherence. This is clinically relevant because long-term endocrine therapy requires sustained treatment exposure, and adverse symptoms can contribute to treatment discontinuation. At present, however, there is insufficient evidence to conclude that an engineered probiotic consortium can reliably increase endoxifen exposure or improve tamoxifen efficacy. Future studies should therefore evaluate microbiome composition, microbial metabolic pathways, tamoxifen and endoxifen pharmacokinetics, treatment adherence, and clinical outcomes simultaneously (50). Aromatase inhibitors, including anastrozole, letrozole, and exemestane, reduce peripheral estrogen synthesis and are widely used in postmenopausal ER-positive breast cancer. Their efficacy therefore depends on sustained suppression of estrogen production rather than direct microbial activation. The microbiome may nevertheless influence the endocrine environment indirectly through estrogen metabolism, systemic inflammation, metabolic signaling, and host physiology. Whether specific microbial configurations modify aromatase-inhibitor pharmacokinetics or therapeutic response remains uncertain. Engineered probiotic consortia could theoretically be designed to modify microbial metabolic functions associated with estrogen recycling or inflammatory signaling. However, such interventions should not be assumed to enhance aromatase-inhibitor efficacy until prospective clinical studies demonstrate a clinically meaningful effect (43).

Endocrine resistance is a major challenge in ER-positive breast cancer. Resistance can arise through multiple mechanisms, including alterations in estrogen-receptor signaling, activation of growth-factor pathways, changes in tumor-cell metabolism, and acquired genomic alterations. The microbiome may potentially contribute indirectly to this process through microbial metabolites and systemic inflammatory signaling. Microbial products can influence host cellular pathways involved in metabolism, immune regulation, and epigenetic signaling. These mechanisms raise the possibility that microbiome-derived signals could influence the tumor microenvironment and endocrine responsiveness. Nevertheless, evidence linking specific microbial species or metabolites directly to clinical endocrine resistance remains insufficient. Future research should therefore avoid interpreting microbiome associations as established mechanisms of endocrine resistance without mechanistic and prospective validation (44). The microbiome–endocrine therapy relationship may differ substantially according to menopausal status. Premenopausal and postmenopausal patients have different sources and concentrations of endogenous estrogen and may receive different endocrine treatment strategies. Consequently, future microbiome studies should stratify participants according to menopausal status and, where relevant, ovarian suppression therapy. Estrogen metabolite profiling should be incorporated alongside microbiome sequencing to determine whether microbial alterations correspond to clinically meaningful changes in hormone metabolism (45). The microbiome–endocrine interface should also be studied alongside tumor molecular characteristics. ER expression level, progesterone-receptor status, HER2 status, acquired ESR1 alterations, PI3K/AKT/mTOR pathway abnormalities, and other molecular features may influence endocrine responsiveness and could potentially interact with host–microbiome signals.

### 5.4 Translational and clinical implications

The integration of engineered probiotic consortia into supportive oncology care offers significant translational and clinical potential, particularly for breast cancer patients undergoing chemotherapy. By targeting the gut microbiome—a central mediator of mucosal integrity, immune regulation, and systemic inflammation—these interventions can address both the local and systemic toxicities that limit treatment efficacy and patient quality of life (46). From a translational perspective, rationally designed consortia allow for precision-based modulation of the microbiome. Multi-omics profiling, including metagenomics, metabolomics, and immunoprofiling, can identify patient-specific microbial deficits and functional disruptions caused by chemotherapy. This information can guide the selection or engineering of consortia that restore key metabolic pathways, enhance short-chain fatty acid production, reinforce epithelial barrier integrity, and modulate inflammatory responses. Such personalized approaches ensure that interventions are tailored to individual host–microbiome interactions, maximizing efficacy while minimizing potential risks (47).

Clinically, probiotic consortia have the potential to reduce the severity and duration of chemotherapy-induced mucositis, diarrhea, and other gastrointestinal complications. By maintaining microbial diversity and preventing pathobiont overgrowth, consortia may reduce infection risk and systemic inflammation, supporting hematologic and immune resilience. Improved gastrointestinal function and decreased systemic toxicity may in turn allow for better adherence to chemotherapy regimens, fewer dose reductions, and more consistent therapeutic exposure, ultimately improving treatment outcomes (48). Furthermore, probiotic consortia may complement existing supportive care strategies, including dietary interventions, pharmacologic antiemetics, and hydration protocols. When combined with synbiotic approaches—prebiotics that enhance microbial growth and metabolite production—these consortia can provide synergistic benefits, enhancing colonization and functional output while maintaining microbial stability under the perturbative effects of chemotherapy (49). Translational challenges remain, including the need for standardized formulations, quality control, regulatory oversight, and rigorous safety evaluation, especially in immunocompromised patients. Clinical trials specifically targeting breast cancer populations are limited, and long-term outcomes, optimal dosing schedules, and strain-specific effects require further investigation. Nevertheless, early-phase studies demonstrate that engineered consortia are generally safe, feasible, and capable of producing measurable biological effects, supporting continued exploration in larger, controlled clinical trials (50) (Table 3).

**TABLE 3:** Probiotic consortia studies.

Study type	Cancer setting	Formulation type	Main findings	Key caveat
Preclinical	Murine chemotherapy-injury models	Selected *Lactobacillus* and bifidobacterium strains	Reduced epithelial apoptosis, preserved tight junctions, lowered inflammatory signaling	Effects were strain-specific and context-dependent (46)
Clinical RCT	Postoperative colorectal cancer on chemotherapy	Probiotic combination	Reduced GI complications, especially diarrhea, increased bacterial diversity, restored taxa shifts, increased SCFAs	Single-course postoperative setting limits generalization (47)
Meta-analysis	Gastrointestinal cancers	Probiotics or synbiotics	Lower nausea/vomiting and diarrhea incidence; increased bifidobacteria and reduced *E. coli*	Pooled studies remained formulation-heterogeneous (48)
Meta-analysis	Mixed oncology populations	Oral probiotics	Lower all-grade and severe diarrhea and oral mucositis; more species associated with greater benefit	Benefit was significant mainly in asian patients (49)
Pilot clinical study	Breast cancer during chemotherapy	7-Strain probiotic formulation	Preliminary improvements in fatigue, nausea, Karnofsky score, and blood urea nitrogen	Small preliminary study needing confirmation (50)

## 6 Gut-directed microbial therapy *versus* breast-tissue-directed microbial delivery

The therapeutic concept of engineered probiotic consortia requires a clear distinction between gut-directed microbial therapy and the substantially more complex strategy of direct breast-tissue-directed microbial delivery. Although both approaches seek to exploit microbiome biology to improve breast cancer treatment, they differ considerably in biological rationale, delivery requirements, safety profile, and translational maturity. At present, gut-directed intervention represents the more plausible and experimentally accessible strategy, whereas deliberate manipulation of the breast-tissue microbiome remains an emerging research concept.

### 6.1 Gut-directed microbial therapy

The principal rationale for gut-directed microbial therapy is that the intestinal microbiome functions as an important regulator of systemic metabolism, epithelial integrity, immune activity, inflammation, and xenobiotic processing. Chemotherapy can disrupt this ecosystem through direct effects on intestinal epithelial cells, alterations in microbial abundance, changes in nutrient availability, and indirect effects arising from antibiotics, hospitalization, dietary changes, and other supportive treatments. Such dysbiosis may contribute to gastrointestinal toxicity and potentially influence systemic treatment responses. Engineered probiotic consortia could therefore be administered with the objective of modifying specific microbial functions rather than simply increasing bacterial diversity. Potential functions include supporting epithelial barrier integrity, producing beneficial microbial metabolites, modulating excessive inflammatory signaling, and potentially altering selected microbial pathways involved in drug metabolism. The major advantage of this approach is that the gastrointestinal tract is naturally accessible to orally administered microbial therapeutics. Potential delivery formats include defined oral microbial formulations, encapsulated preparations, and other gastrointestinal-targeted formulations designed to improve stability and release at appropriate intestinal sites. However, successful administration does not necessarily require permanent colonization. A transiently active microbial consortium may be sufficient if it produces a desired biological effect during the period of chemotherapy-associated vulnerability [51, 52].

### 6.2 Breast-tissue-directed microbial delivery

The breast tissue microbiome represents a fundamentally different therapeutic target. Increasing evidence suggests that microbial communities associated with breast tissue and the local tumor microenvironment may participate in tumor biology, immune regulation, metabolism, and therapeutic response. However, the biological significance, composition, stability, and spatial distribution of these microbial communities remain incompletely defined. Directly introducing engineered microorganisms into breast tissue would therefore involve substantially greater uncertainty. Potential approaches might theoretically include localized delivery associated with a tumor-directed procedure or other future tissue-targeting technologies. However, these approaches remain experimental and should not currently be considered established clinical strategies. The principal concern is that deliberate introduction of live engineered microorganisms into tumor or normal breast tissue could result in uncontrolled persistence, local inflammation, infection, tissue dissemination, or unexpected interactions with tumor and immune cells. These risks may be particularly important in patients receiving cytotoxic chemotherapy or other treatments that compromise immune function [53, 54].

## 7 Lifestyle, diet, and microbiome-supportive strategies

The development of engineered probiotic consortia should not be viewed as a purely molecular or genetic intervention. The intestinal microbiome is continuously shaped by diet, nutritional status, physical activity, medication exposure, antibiotic use, sleep, environmental factors, and other host behaviors. Consequently, the effectiveness and safety of an engineered microbial consortium may depend partly on the ecological environment into which it is introduced. A comprehensive microbiome-supportive strategy should therefore combine appropriately selected microbial therapeutics with evidence-based lifestyle and nutritional measures rather than treating engineered probiotics as an isolated intervention.

### 7.1 Dietary modulation

Diet is among the strongest modifiable determinants of intestinal microbial composition and function. Dietary patterns influence microbial diversity, fermentation, metabolite production, intestinal barrier integrity, and inflammatory signaling. Adequate intake of fiber-containing foods can provide substrates for microbial fermentation and support production of short-chain fatty acids, including acetate, propionate, and butyrate. These metabolites may contribute to epithelial energy metabolism, mucosal integrity, and immune regulation. For breast cancer patients receiving chemotherapy, however, dietary recommendations must be individualized. Patients experiencing diarrhea, mucositis, nausea, vomiting, abdominal pain, or significant gastrointestinal intolerance may temporarily require modifications in food texture, fiber type, meal frequency, or other dietary components. Conversely, unnecessarily restrictive diets may reduce nutritional adequacy and potentially deprive beneficial microorganisms of fermentable substrates. The objective should therefore be nutritional adequacy and gastrointestinal tolerance rather than indiscriminate dietary restriction. Dietary diversity may also be relevant because different plant-derived foods provide distinct substrates for microbial metabolism. Where clinically tolerated, a varied diet containing appropriate fruits, vegetables, whole grains, legumes, and other nutrient-dense foods may support a more functionally diverse microbial ecosystem. Nevertheless, dietary interventions should be guided by the patient’s nutritional status, treatment regimen, gastrointestinal symptoms, cultural dietary practices, and clinical requirements rather than by a universal “microbiome diet.”

### 7.2 Nutritional optimization during chemotherapy

Malnutrition and inadequate dietary intake can independently worsen treatment tolerance and clinical outcomes. Consequently, nutritional optimization should form an important component of microbiome-supportive care. Assessment should include dietary intake, weight trajectory, nutritional risk, gastrointestinal symptoms, and relevant biochemical or functional indicators where clinically appropriate. The potential interaction between nutritional support and engineered probiotic consortia is particularly important. A consortium may require specific substrates to perform its intended metabolic functions, meaning that its efficacy could be influenced by the patient’s dietary environment. Future clinical trials should therefore document nutritional intake and evaluate whether specific dietary patterns enhance or diminish microbial therapeutic activity.

### 7.3 Physical activity

Appropriately prescribed physical activity may influence systemic inflammation, metabolic health, gastrointestinal function, and overall treatment tolerance. Although the direct effect of physical activity on engineered probiotic consortia remains insufficiently established, maintaining physical activity within individual clinical limits may contribute to a favorable host environment. Recommendations should be individualized according to cancer treatment, anemia, fatigue, cardiovascular status, musculoskeletal limitations, and overall functional capacity. Physical activity should be considered a supportive component of comprehensive cancer care rather than a direct replacement for microbiome-directed therapy.

### 7.4 Antibiotic stewardship

Antibiotic exposure is one of the most important clinical factors capable of disrupting intestinal microbial ecosystems. Antibiotics may reduce microbial diversity, alter metabolic pathways, and potentially interfere with colonization or function of administered microbial therapeutics. Unnecessary antibiotic exposure should therefore be avoided, while clinically indicated antibiotics should never be withheld when treatment of suspected or confirmed infection is necessary. Future clinical studies of engineered probiotic consortia should systematically document antibiotic type, duration, timing relative to microbial therapy, and indication. These variables may be important determinants of treatment response and should be incorporated into microbiome-based predictive models.

### 7.5 Medication-associated microbiome effects

Other commonly used medications can also influence the intestinal microbiome. These may include proton-pump inhibitors, corticosteroids, antiemetics, analgesics, laxatives, antidiarrheal medications, and antimicrobial agents. Breast cancer patients frequently receive several supportive medications simultaneously, making medication-related microbial effects an important potential confounder. Future microbiome studies should therefore characterize concomitant medication exposure and assess whether specific medications modify the activity or safety of engineered microbial consortia.

### 7.6 Hydration and gastrointestinal support

Adequate hydration is particularly important in patients experiencing diarrhea, vomiting, reduced oral intake, or other gastrointestinal complications. Appropriate management of dehydration and electrolyte disturbances should remain part of standard supportive oncology care. Engineered probiotics should complement established clinical management rather than replace evidence-based interventions such as appropriate fluid and electrolyte replacement, nutritional support, antiemetic therapy, or treatment of documented infections.

### 7.7 Sleep, stress, and behavioral factors

Sleep quality and psychosocial stress may influence immune function, metabolism, gastrointestinal physiology, and health behaviors, although the specific relationship between these factors and engineered probiotic efficacy remains incompletely characterized. Future studies should consider these variables when evaluating longitudinal microbiome changes and patient-reported outcomes. However, claims that specific behavioral interventions can directly “reset” the microbiome or prevent chemotherapy toxicity should be avoided until supported by appropriate clinical evidence.

### 7.8 Integration with engineered probiotic consortia

The most appropriate future model is therefore not probiotic therapy *versus* lifestyle modification, but an integrated microbiome-supportive framework: Standard breast cancer treatment → nutritional optimization → appropriate dietary pattern → physical activity as tolerated → rational medication and antibiotic use → engineered probiotic consortium when clinically indicated → longitudinal microbiome and toxicity monitoring.

Within this framework, diet and lifestyle provide the ecological context in which an engineered consortium operates. The microbial intervention can then be designed according to the patient’s specific microbiome and metabolic deficiencies rather than administered independently of the surrounding host environment.

### 7.9 Precision nutrition and microbial engineering

An important future direction is the development of precision nutrition–microbiome combinations. Metagenomic and metabolomic profiling could identify microbial functions that are deficient in an individual patient, while dietary assessment could determine whether appropriate substrates are available to support restoration of those functions. For example, if a patient demonstrates reduced capacity for production of beneficial microbial metabolites, a future intervention might combine a defined microbial consortium with an individualized nutritional strategy designed to provide appropriate substrates. Such an approach would require prospective validation and should not currently be interpreted as established clinical practice.

### 7.10 Avoiding overpromotion and unsupported claims

Because engineered probiotic consortia remain an emerging technology, promotion should emphasize evidence-based supportive oncology rather than commercial probiotic supplementation. Patients should be informed that commercially available probiotic products are not equivalent to rigorously characterized engineered microbial therapeutics and that product composition, potency, and clinical evidence can vary considerably. Healthcare professionals should also avoid presenting dietary changes, supplements, or probiotics as substitutes for chemotherapy or other established breast cancer treatments. The appropriate objective is to complement evidence-based cancer treatment while improving treatment tolerance and quality of life.

## 8 Future correlative and multi-omic assessments

Future research should move beyond conventional characterization of gut microbial composition and investigate the functional, metabolic, immunological, pharmacological, and clinical consequences of microbiome manipulation during breast cancer chemotherapy. Although taxonomic profiling can identify changes in bacterial abundance and diversity, it does not necessarily establish whether those organisms are metabolically active, causally involved in toxicity, or responsive to an engineered probiotic consortium. A comprehensive multi-omic framework could therefore provide a more precise understanding of how engineered microbial communities influence chemotherapy tolerance and treatment outcomes.

### 8.1 Longitudinal microbiome profiling

Future studies should obtain serial microbiome samples rather than relying exclusively on a single pretreatment measurement. Stool sampling before chemotherapy, during individual treatment cycles, at the onset of toxicity, after probiotic intervention, and during post-treatment recovery could establish temporal relationships between chemotherapy exposure, microbiome disruption, and clinical toxicity. Shotgun metagenomic sequencing could characterize microbial species and functional genes with greater resolution than 16S rRNA sequencing. Longitudinal analysis could determine whether engineered consortia successfully establish their intended functional effects, whether they alter indigenous microbial communities, and whether microbiome recovery occurs after completion of chemotherapy. Particular attention should be given to microbial diversity, ecological stability, pathogenic or opportunistic organism expansion, antimicrobial-resistance genes, and functional pathways associated with drug metabolism and host immune regulation.

### 8.2 Metatranscriptomic and functional microbiome assessment

Metagenomic data describe the functional potential of microorganisms, whereas metatranscriptomics can determine which microbial genes and pathways are actively expressed. This distinction is particularly important for engineered probiotic consortia because the presence of an engineered organism does not necessarily demonstrate that its intended biological function is occurring *in vivo*. Metatranscriptomic assessment could therefore determine whether engineered strains activate their therapeutic pathways under chemotherapy-associated gastrointestinal conditions. These analyses could be integrated with microbial proteomics where feasible to establish whether predicted functional pathways ultimately result in biologically active proteins or enzymes.

### 8.3 Microbiome metabolomics

Microbial metabolites represent an important bridge between intestinal microorganisms and host physiology. Future studies should evaluate short-chain fatty acids, bile-acid derivatives, tryptophan metabolites, polyamines, indole compounds, and other microbial products that may influence epithelial integrity, immune signaling, inflammation, and systemic metabolism. Metabolomic profiling could identify metabolic signatures associated with severe chemotherapy toxicity and determine whether engineered consortia restore depleted or altered metabolites. Metagenomics–metabolomics integration may be particularly informative because it can connect specific microbial functional pathways with measurable biochemical consequences.

### 8.4 Intestinal barrier and mucosal biomarkers

Because gastrointestinal epithelial injury is a principal target of the proposed intervention, future trials should incorporate objective measures of intestinal barrier function. Potential assessments include circulating or fecal markers associated with epithelial injury and permeability, together with inflammatory and mucosal biomarkers. Combining these measurements with microbiome and metabolomic profiles could help determine whether clinical improvement reflects genuine restoration of intestinal barrier function rather than nonspecific symptomatic relief.

### 8.5 Host immune and inflammatory profiling

The microbiome interacts closely with innate and adaptive immunity. Future studies should therefore evaluate inflammatory cytokines, chemokines, immune-cell populations, and relevant immune activation pathways before and after microbiome intervention. This is particularly important in breast cancer because some patients receive therapies in which immune responses contribute substantially to therapeutic efficacy. The objective should be to determine whether engineered probiotic consortia reduce pathological treatment-associated inflammation without suppressing beneficial antitumor immunity. Potential analyses could include circulating immune-cell phenotyping, inflammatory cytokine panels, markers of innate immune activation, and selected T-cell functional parameters.

### 8.6 Tumor genomic and epigenomic correlation

Microbiome data should eventually be integrated with tumor molecular characteristics. Tumor sequencing could identify associations between microbiome phenotypes and alterations involving BRCA1/BRCA2, TP53, PIK3CA, HER2-related pathways, hormone-receptor signaling, DNA-repair pathways, and other clinically relevant molecular features. Epigenomic profiling could additionally investigate DNA methylation, chromatin-associated changes, and non-coding RNA networks potentially influenced by systemic inflammatory or metabolic signals originating from the microbiome. These analyses should not imply that engineered probiotics directly correct tumor mutations or epigenetic abnormalities. Rather, they could determine whether particular molecular breast cancer phenotypes are associated with distinct microbiome configurations, treatment responses, or toxicity patterns.

### 8.7 Pharmacokinetic and pharmacodynamic correlation

An essential future research priority is determining whether engineered microbial consortia modify anticancer drug disposition. Microorganisms can metabolize xenobiotics and potentially influence drug absorption, transformation, or elimination. Clinical studies should therefore integrate microbiome profiling with pharmacokinetic measurements of relevant chemotherapy agents and metabolites. Pharmacodynamic analyses could subsequently determine whether microbial intervention changes drug exposure, treatment activity, or toxicity. This is particularly important for engineered organisms deliberately designed to modify microbial metabolism because unintended alteration of chemotherapy pharmacokinetics could reduce therapeutic efficacy.

### 8.8 Host pharmacogenomics and microbiome interactions

Host genetic variation may interact with microbial metabolism to influence chemotherapy toxicity. Future studies could therefore integrate pharmacogenomic information with microbiome data to distinguish host-derived and microbiome-derived determinants of treatment response. A combined model incorporating host genotype, tumor genotype, microbial functional capacity, drug exposure, and clinical phenotype may ultimately provide better toxicity prediction than any individual data source.

### 8.9 Nutritional and dietary correlation

Diet is a major determinant of microbial ecology and should be incorporated into future studies rather than treated as an uncontrolled confounder. Detailed dietary assessment could determine whether fiber intake, dietary diversity, nutritional adequacy, and other dietary variables modify microbiome composition or response to an engineered consortium. Nutritional status, weight trajectory, gastrointestinal symptoms, and biochemical indicators of nutritional adequacy should also be monitored. This could help distinguish the effects of the engineered consortium from those produced by dietary changes occurring during chemotherapy.

### 8.10 Medication and antibiotic exposure

Antibiotics can substantially alter the intestinal microbiome and may influence the activity of microbial therapeutics. Future studies should therefore systematically record antibiotic exposure, corticosteroids, proton-pump inhibitors, antiemetics, analgesics, laxatives, antidiarrheal agents, and other medications that may influence gastrointestinal physiology or microbial composition. This information should be incorporated into statistical models because treatment-associated microbiome changes may otherwise be incorrectly attributed to the engineered probiotic consortium.

### 8.11 Patient-reported outcomes and digital phenotyping

Molecular measurements should be correlated with patient-centered outcomes. Serial assessment of diarrhea, abdominal symptoms, nausea, appetite, fatigue, functional status, treatment burden, and health-related quality of life can establish whether biological microbiome changes translate into meaningful clinical benefits. Digital symptom monitoring and electronic patient-reported outcomes could facilitate high-frequency assessment between chemotherapy cycles and allow early identification of patients developing clinically important toxicity.

### 8.12 Correlation with treatment delivery and oncological outcomes

Future investigations should determine whether microbiome stabilization translates into improved delivery of planned cancer therapy. Important endpoints include dose intensity, treatment delays, dose reductions, hospitalization, premature treatment discontinuation, and completion of the intended chemotherapy regimen. These outcomes should be evaluated alongside pathological response, objective response, progression-free survival, and overall survival. A successful microbiome intervention should ideally reduce toxicity while maintaining or improving cancer control.

## 9 Conclusion

Chemotherapy remains a cornerstone of breast cancer therapy, yet its clinical utility is frequently limited by gastrointestinal toxicity, systemic inflammation, and treatment-related complications that compromise patient quality of life and adherence. Emerging evidence underscores the central role of the gut microbiome in mediating these toxicities, revealing that chemotherapy-induced dysbiosis exacerbates mucosal injury, impairs epithelial repair, and amplifies systemic inflammatory responses. Traditional single-strain probiotics have shown limited efficacy in addressing this multifactorial disruption, highlighting the need for more sophisticated, multi-functional interventions.

Engineered probiotic consortia represent a promising strategy to restore microbial balance, enhance mucosal barrier integrity, modulate immune responses, and support metabolic homeostasis. By combining multiple strains with complementary metabolic and immunomodulatory functions, consortia can provide synergistic protection against chemotherapy-induced mucositis and systemic toxicity. Advances in synthetic biology, computational modeling, and targeted delivery technologies have enabled the rational design of these consortia, allowing for precision-guided interventions tailored to patient-specific microbiome profiles, chemotherapy regimens, and immune status. Preclinical studies demonstrate that multi-strain consortia reduce inflammatory signaling, restore short-chain fatty acid production, and preserve epithelial integrity, while early clinical evidence suggests improved chemotherapy tolerance and reduced gastrointestinal toxicity. Despite challenges related to standardization, safety evaluation, and regulatory oversight, engineered probiotic consortia hold significant potential to transform supportive care in breast cancer, offering a biologically grounded, mechanism-based approach to mitigating treatment-related toxicity.
